# Supplementary material for: Gene drive for population genetic control: non-functional resistance and parental effects
Source: Proc Biol Sci. 2019 Oct 30;286(1914):20191586. doi: 10.1098/rspb.2019.1586 (PMC6842858; doi:10.1098/rspb.2019.1586)
Supplement: Supplementary Modelling Information [file rspb20191586supp1.pdf]

Supplementary Model Description

Population Genetics Model

We use discrete-generation recursion equations for the genotype frequencies [1], treating males and females separately as in Kyrou et al. [2]. We consider three alleles, W (wildtype), D (driver), and R (non-functional resistant), and therefore six basic genotypes where $F_{\mathrm{ij}}(t)$ and $M_{\mathrm{ij}}(t)$ are frequencies of genotypes i/j of females in the female population and males in the male population. The resistant allele causes a change in the target sequence such that it is no longer recognised by the nuclease, and function of the gene is destroyed in R alleles.

Modelling parental deposition. We consider that in addition to cleavage and repair of the W allele during meiosis, further cleavage/repair can occur in the embryo if deposited nuclease is present from gametes derived from one or both transgenic parents. Previously, embryonic end-joining effects (maternal only) were modelled as acting immediately in the zygote [1, 3]. Here, we assume that during mosquito development, parentally supplied enzyme in the embryo continues to be active in cells from the zygote stage up until the point where the germline stem cells are established in the gonads. After this time, parental nuclease is considered not to be active and gene transmission in gametes from a germline cell is according to its own genotype and nuclease if drive is present. Individuals therefore may end up mosaic in their soma, affecting female fitness (as modelled in Kyrou et al. [2]) and mosaic in their germline cells which alters gene transmission, as modelled here. We assume no correlation between the effect on fitness and the effect on gene transmission. There are six zygotic genotypes (i/j = W/W, W/D, W/R, D/D, D/R, R/R). To take into account effects on fitness and gene transmission in the embryo due to parental deposition, we distinguish frequencies of individuals with a W allele (W/W, W/D, W/R) by whether they have a transgenic mother, father or both transgenic parents, using superscripts a =10, 01 or 11 (e.g., $F_{\mathrm{ij}}^{10}(t)$ is a female that started as a zygote i/j with effects in the embryo from maternal nuclease). Note that frequencies $F_{\mathrm{WW}}(t)$ and $F_{\mathrm{WD}}(t)$ without a superscript denote individuals that have not received any parental nuclease from either parent. Differentiating adults by parentage to account for embryonic effects therefore leads to a total of fourteen genotypes: two that did not have transgenic parents (W/W, W/R), nine with a transgenic mother, father or both parents $(\mathrm{W/W}, \mathrm{W/D}, \mathrm{W/R})^{a}$, with $a = 01, 10, \text{or } 11$, that may be mosaic in either their soma or germline, and three for which it is not necessary to keep track of parentage (D/D, D/R, R/R) since there are no W alleles present.

Fitness. Let $w_{\mathrm{ij}} \leq 1$ represent the fitness of genotype $\mathrm{i/j}$ relative to $w_{\mathrm{WW}} = 1$ for the wild-type homozygote. We assume no fitness effects in males. Fitness effects in females are manifested as differences in the relative ability of genotypes to participate in mating and reproduction. We assume the target gene is needed for female participation in reproduction, thus D/D, D/R and R/R females do not reproduce ($w_{\mathrm{DD}} = w_{\mathrm{DR}} = w_{\mathrm{RR}} = 0$). For a haplosufficient gene, there is no reduction in fertility in females from having only one copy of the gene. To model parental effects on fitness, W/X (X = W, D, R) genotypes with parental nuclease are assigned a reduced fitness w_{WX}^{10}, w_{WX}^{01}, or w_{WX}^{11} depending on whether the nuclease was derived from a transgenic mother, father, or both. We assume that parental effects are the same whether the parent(s) had one or two drive alleles. For simplicity, the same baseline reduced fitness of w^{10}, w^{01}, w^{11}, is assigned to all genotypes of type W/X (X = W, D, R). To model effects of leaky expression on female fitness with no parental effects, all W/D females regardless of parentage are assigned fitness w ($w_{\mathrm{WD}}^{10}, w_{\mathrm{WD}}^{01}, w_{\mathrm{WD}}^{11} = w$), with no fitness reduction in other genotypes. For the case of a haploinsufficient gene and no parental effects, all W/D and W/R genotypes are

assigned reduced fitness w ($w_{\mathrm{WD}}^{10}, w_{\mathrm{WD}}^{01}, w_{\mathrm{WD}}^{11}, w_{\mathrm{WR}}^{10}, w_{\mathrm{WR}}^{01}, w_{\mathrm{WR}}^{11}, w_{\mathrm{WR}} = w$), with no fitness reduction in other genotypes.

Gene transmission

Embryonic expression. Effects on gene transmission are due to parental deposition in the embryo are modelled as follows. Genotypes W/X (X = W, D, R) with parental nuclease will end up mosaic in their germline stem cells depending on the amount of cleavage and embryonic EJ (end-joining) or HR (homologous repair) due to nuclease from mother, father or both. We assume that effects of parental nuclease are the same in males and females. The proportion of wild-type alleles that are cleaved and repaired to resistant alleles by end joining in the embryo is δ_{e}^{10}, δ_{e}^{01}, δ_{e}^{11} with nuclease from mother, father or both; the proportion cleaved and repaired by embryonic HR is e_{e}^{10}, e_{e}^{01}, e_{e}^{11} with nuclease from mother, father or both. Table S1 shows the resulting proportions of germline stem cells of different genotypes in gonads W/X (X = W, D, R) mosaics with parental nuclease.

*Meiotic expression***.** Gamete production from germline stem cells is then according to its own genotype (and its own nuclease if the drive construct is present), since we assume that parental nuclease is no longer active after this stage (see Fig. 1). Germline W/D cells produce gametes at meiosis in the ratio W: D: R as follows:

$\frac{(1-e_{\mathrm{m}}^{\mathrm{F}}-\delta_{\mathrm{m}}^{\mathrm{F}})}{2} : \frac{(1+e_{\mathrm{m}}^{\mathrm{F}})}{2} : \frac{\delta_{\mathrm{m}}^{\mathrm{F}}}{2}$ in females

$\frac{(1-e_{\mathrm{m}}^{\mathrm{M}}-\delta_{\mathrm{m}}^{\mathrm{M}})}{2} : \frac{(1+e_{\mathrm{m}}^{\mathrm{M}})}{2} : \frac{\delta_{\mathrm{m}}^{\mathrm{M}}}{2}$ in males

Here $\delta_{\mathrm{m}}^{\mathrm{F}}$ and $\delta_{\mathrm{m}}^{\mathrm{M}}$ are rates of meiotic end-joining repair to resistant (R) alleles during meiosis and $e_{\mathrm{m}}^{\mathrm{F}}$ and $e_{\mathrm{m}}^{\mathrm{M}}$ are homing rates (HR repair) of the driver allele in females and in males (for baseline parameters, we assume that rates are the same in both sexes: $\delta_{\mathrm{m}}^{\mathrm{F}} = \delta_{\mathrm{m}}^{\mathrm{M}} = \delta_{\mathrm{m}}$ and $e_{\mathrm{m}}^{\mathrm{F}} = e_{\mathrm{m}}^{\mathrm{M}} = e_{\mathrm{m}}$). For all other genotypes, inheritance is Mendelian.

Recursion equations. We consider the gamete contributions from each genotype, including parental effects on fitness and gene transmission. In addition to W and R gametes that are derived from parents that have no drive allele and therefore have no deposited nuclease, gametes from transgenic females and males carry nuclease that is transmitted to the zygote, and these are denoted as W*, D*, R*. The proportions of eggs $e_i(t)$ with allele $i = \mathrm{W, R, W^*, D^*, R^*}$ produced by females participating in reproduction are given in terms of the female genotype frequencies:

$$E_i(t) = \frac{\sum_{k=1}^{14} c_{k,i}^{F} w_k F_k(t)}{\sum_{k=1}^{14} w_k F_k(t)} \quad (1)$$

where k is summed such that {1,2,3…14} correspond to the fourteen genotypes k, some of which are differentiated by parentage as described above. Note that the denominator in Eq. (1) is the average female reproductive fitness, calculated as the sum of the female frequencies in the female population times their fitnesses:

$$\bar{w}_f(t) = \sum_{k=1}^{14} w_k F_k(t) \quad (2)$$

The coefficients $c_{k,i}^{F}$ in (1a) correspond to the proportion of the gametes from female individuals of type k that carry allele $i = \mathrm{W, R, W^*, D^*, R^*}$ and are shown in Table S2, with rows corresponding to genotype k and columns to allele i. Similarly for sperm:

$$S_i(t) = \frac{\sum_{k=1}^{14} c_{k,i}^{M} w_k M_k(t)}{\sum_{k=1}^{14} w_k M_k(t)}$$

where for males, $\sum_{k=1}^{14} w_k M_k(t) = 1$ since we assume no fitness reduction. We start with an equal number of males and females, with an initial frequency of wildtype females in the female population of $F_{\mathrm{WW}}(t=0) = 1$, wildtype males in the male population of $M_{\mathrm{WW}}(t=0) = 0.99$, and $M_{\mathrm{WD}}^{01}(t=0) = 0.01$ heterozygote drive males that inherited the drive from their mothers. Assuming a 50:50 ratio of males and females in progeny, after the starting generation, genotype frequencies the next generation are the same in males and females, and are given by the recursion relations, with $F_{\mathrm{k}}(t+1) = M_{\mathrm{k}}(t+1)$. Both are given by $G_{\mathrm{k}}(t+1)$ in the following set of equations (as in [2]) in terms of the gamete proportions in the previous generation, assuming random mating:

$$G_{\mathrm{WW}}(t+1) = E_{\mathrm{W}}(t) S_{\mathrm{W}}(t)$$

$$G_{\mathrm{WW}}^{10}(t+1) = E_{\mathrm{W^*}}(t) E_{\mathrm{W}}(t)$$

$$G_{\mathrm{WW}}^{01}(t+1) = E_{\mathrm{W}}(t)\, S_{\mathrm{W^*}}(t)$$

$$G_{\mathrm{WW}}^{11}(t+1) = E_{\mathrm{W^*}}(t)\, S_{\mathrm{W^*}}(t)$$

$$G_{\mathrm{WD}}^{10}(t+1) = E_{\mathrm{D^*}}(t) S_{\mathrm{W}}(t)$$

$$G_{\mathrm{WD}}^{01}(t+1) = E_{\mathrm{W}}(t)\, S_{\mathrm{D^*}}(t)$$

$$G_{\mathrm{WD}}^{11}(t+1) = E_{\mathrm{W^*}}(t)\, S_{\mathrm{D^*}}(t) + E_{\mathrm{D^*}}(t) S_{\mathrm{W^*}}(t)$$

$$G_{\mathrm{WR}}(t+1) = E_{\mathrm{W}}(t) S_{\mathrm{R}}(t) + E_{\mathrm{R}}(t) S_{\mathrm{W}}(t)$$

$$G_{\mathrm{WR}}^{10}(t+1) = E_{\mathrm{W^*}}(t)\, S_{\mathrm{R}}(t) + E_{\mathrm{R^*}}(t) S_{\mathrm{W}}(t)$$

$$G_{\mathrm{WR}}^{01}(t+1) = E_{\mathrm{W}}(t)\, S_{\mathrm{R^*}}(t) + E_{\mathrm{R}}(t) S_{\mathrm{W^*}}(t)$$

$$G_{\mathrm{WR}}^{11}(t+1) = E_{\mathrm{W^*}}(t)\, S_{\mathrm{R^*}}(t) + E_{\mathrm{R^*}}(t) S_{\mathrm{W^*}}(t)$$

$$G_{\mathrm{DD}}(t+1) = E_{\mathrm{D^*}}(t)\, S_{\mathrm{D^*}}(t)$$

$$G_{\mathrm{DR}}(t+1) = \big(E_{\mathrm{R}}(t) + E_{\mathrm{R^*}}(t)\big) S_{\mathrm{D^*}}(t) + E_{\mathrm{D^*}}(t)\big(S_{\mathrm{R}}(t) + S_{\mathrm{R^*}}(t)\big)$$

$$F_{\mathrm{RR}}(t+1) = \big(E_{\mathrm{R}}(t) + E_{\mathrm{R^*}}(t)\big)\big(S_{\mathrm{R}}(t) + S_{\mathrm{R^*}}(t)\big)$$

Load. Assuming no effects on male fertility, and given an equal sex ratio, the load on the population at time t is defined in terms of the average female fitness in the female population (Eq. 2):

$$L(t) = 1 - 2\, \bar{w}_{\mathrm{f}}(t)$$

It is zero when only wildtypes are present, and one if the drive has fixed and the average female fitness is zero. Increases in load predicted do not predict absolute changes in population density in the field but can be an indication of comparative potential reductions [4]. Complete suppression is predicted to occur in panmictic models when the population growth rate at low density is less than $1/(1-L)$.

All calculations are carried out using Wolfram Mathematica [5].

References

[1] Hammond, A.M., Kyrou K., Bruttini M., North A., Galizi R., Karlsson X., Kranjc N., Carpi F. M., D'Aurizio R., Crisanti A., and T. Nolan. The creation and selection of mutations resistant to a gene drive over multiple generations in the malaria mosquito. *PLoS Genet* **13**, e1007039 (2017).

[2] Kyrou, K., Hammond, A. M., Galizi, R., Kranjc, N., Burt, A., Beaghton, A. K., Nolan, T. and A. Crisanti (2018). A CRISPR-Cas9 gene drive targeting doublesex causes complete population suppression in caged Anopheles gambiae mosquitoes. *Nature Biotechnology* **36**, 11 (2018).

[3] Papathanos, P. A., Windbichler, N., Menichelli, M., Burt, A. and Crisanti, A. The vasa regulatory region mediates germline expression and maternal transmission of proteins in the malaria mosquito *Anopheles gambiae:* a versatile tool for genetic control strategies. *BMC Mol Biol* **10**, 65 (2009).

[4] Deredec, A. Burt, A. and H. C. J. Godfray, The Population Genetics of Using Homing Endonuclease Genes in Vector and Pest Management, *Genetics* **170**, 4 (2008).

[5] Wolfram Research, Inc., 2017 Mathematica 11.2, Champaign, IL.
