# Supplementary material for: Gene drive for population genetic control: non-functional resistance and parental effects
Source: Proc Biol Sci. 2019 Oct 30;286(1914):20191586. doi: 10.1098/rspb.2019.1586 (PMC6842858; doi:10.1098/rspb.2019.1586)
Supplement: Figure S1 [file rspb20191586supp2.pdf]

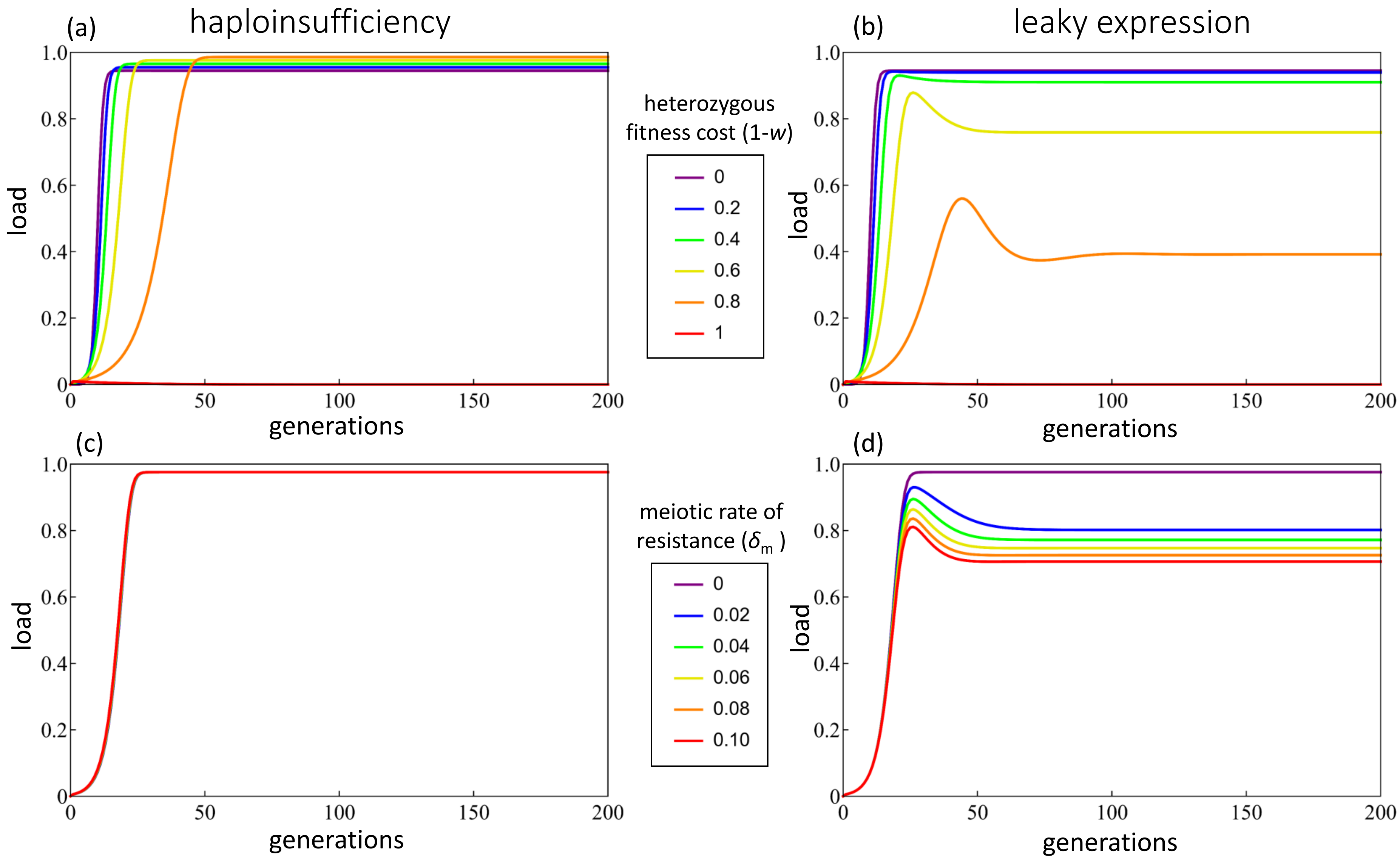

Fig. S1. Dynamics of load over time for different values of the heterozygote fitness cost (a, b; a value of 1 indicates complete sterility) or the rate at which resistant alleles arise (c, d; a value of 0.1 indicates all W alleles not converted to D are repaired by EJ to form R) in the case of partial haplo-insufficiency (a, c) or leaky expression (b, d). In (c) female fitness W/D = W/R = 0.4; and in (d) female fitnesses are W/D = 0.4, W/R = 1. Higher heterozygote fitness costs increase the time taken for the load to reach the equilibrium value for both partial haploinsufficiency (a) and leaky expression (b). For haploinsufficiency (c), increasing the meiotic rate of resistance arising has no effect since drive and resistant alleles have the same fitness costs.
