# Supplementary material for: Gene drive for population genetic control: non-functional resistance and parental effects
Source: Proc Biol Sci. 2019 Oct 30;286(1914):20191586. doi: 10.1098/rspb.2019.1586 (PMC6842858; doi:10.1098/rspb.2019.1586)
Supplement: Figure S2 [file rspb20191586supp3.pdf]

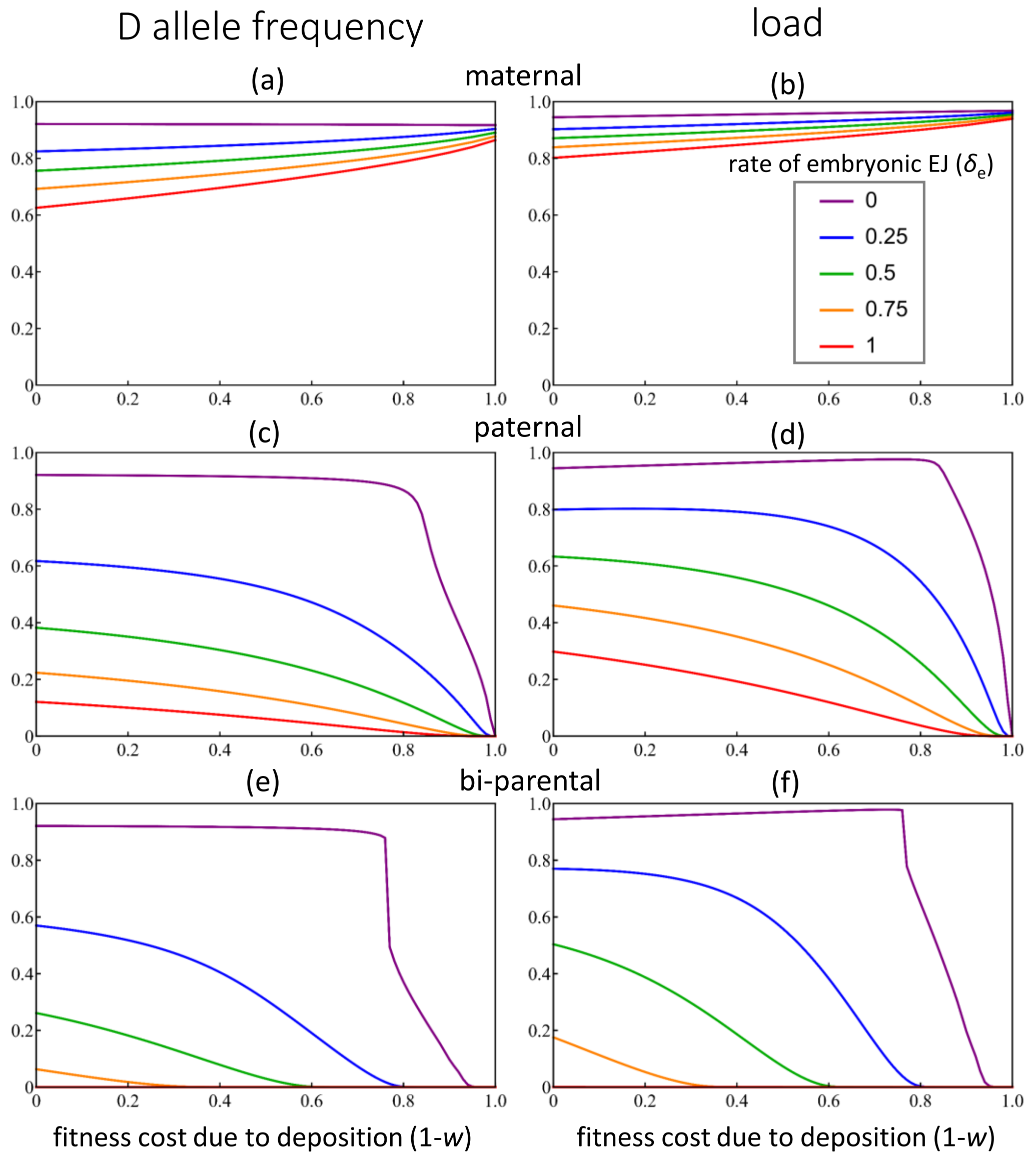

Fig. S2. Equilibrium drive allele frequency (a,c,e) and load (b,d,f) (after 200 generations) when there are parental effects on both fitness and gene transmission, as a function of combined parental effects of reduction in W/D female heterozygote fitness (y-axis) and of rate of resistance from embryonic EJ (different coloured lines). In (a & b), effects are maternal only (w^{10} = w, w^{01} = 1, w^{11} = w; $\delta_e^{10} = \delta_e$, δ_e^{01} = 1, $\delta_e^{11} = \delta_e$), in (c & d) paternal only (w^{10} = 1, w^{01} = *w*, w^{11} =*w*; δ_e^{10} =1, $\delta_e^{01} = \delta_e$, $\delta_e^{11} = \delta_e$), and in (e & f) effects are from both parents assuming w^{11} = w and δ_e^{11} = δ_e. The purple line in each plot (δ_e = 0) is the same as the corresponding line in Fig. 4 of the main text.
