# Supplementary material for: Gene drive for population genetic control: non-functional resistance and parental effects
Source: Proc Biol Sci. 2019 Oct 30;286(1914):20191586. doi: 10.1098/rspb.2019.1586 (PMC6842858; doi:10.1098/rspb.2019.1586)
Supplement: Tables S1, S2, and S3 [file rspb20191586supp4.pdf]

Table S1. Proportions of different types of germline stem cells in female or male mosaic genotypes $G_{\mathrm{ij}}^{a} = \{F_{\mathrm{ij}}^{a}, M_{\mathrm{ij}}^{a}\}$, derived from zygotes of type $\mathrm{i/j} = \mathrm{W/W}, \mathrm{W/D}, \mathrm{W/R}$ with parental nuclease from a mother, father or both, $a = 10, 01,$ or 11.

Genotype	Germline stem cells					
	W/W	W/D	W/R	D/D	D/R	R/R
G_{WW}^{a}	$(1-\delta_{\mathrm{e}}^{a})^2$	0	$2(1-\delta_{\mathrm{e}}^{a})\delta_{\mathrm{e}}^{a}$	0	0	$(\delta_{\mathrm{e}}^{a})^2$
G_{WD}^{a}	0	$1-e_{\mathrm{e}}^{a}-\delta_{\mathrm{e}}^{a}$	0	e_{e}^{a}	δ_{e}^{a}	0
G_{WR}^{a}	0	0	$1-e_{\mathrm{e}}^{a}-\delta_{\mathrm{e}}^{a}$	0	0	$e_{\mathrm{e}}^{a}+\delta_{\mathrm{e}}^{a}$

Table S2. The proportion of each type of gamete (eggs or sperm) produced by the 14 adult types (6 zygotic genotypes, differentiated according to parental effect) and the female fitnesses (male fitnesses are assumed to be one). For parameters e_{m}^{G} and δ_{m}^{G}, the superscript G = F for females and M for males.

Genotype ǂ	Female fitness	Gametes produced				
		W	R	W*	D*	R *
W/W	1	1	0	0	0	0
W/W (10)	w^{10}	$1-\delta_{\mathrm{e}}^{10}$	δ_{e}^{10}	0	0	0
W/W (01)	w^{01}	$1-\delta_{\mathrm{e}}^{01}$	δ_{e}^{01}	0	0	0
W/W (11)	w^{11}	$1-\delta_{\mathrm{e}}^{11}$	δ_{e}^{11}	0	0	0
W/D (10)	w^{10}	0	0	$(1-e_{\mathrm{m}}^{G}-\delta_{\mathrm{m}}^{G})(1-e_{\mathrm{e}}^{10}-\delta_{\mathrm{e}}^{10})/2$	$e_{\mathrm{e}}^{10}+\delta_{\mathrm{e}}^{10}/2+(1+e_{\mathrm{m}}^{G})(1-e_{\mathrm{e}}^{10}-\delta_{\mathrm{e}}^{10})/2$	$\delta_{\mathrm{m}}^{G}(1-e_{\mathrm{e}}^{10}-\delta_{\mathrm{e}}^{10})/2+\delta_{\mathrm{e}}^{10}/2$
W/D (01)	w^{01}	0	0	$(1-e_{\mathrm{m}}^{G}-\delta_{\mathrm{m}}^{G})(1-e_{\mathrm{e}}^{01}-\delta_{\mathrm{e}}^{01})/2$	$e_{\mathrm{e}}^{01}+\delta_{\mathrm{e}}^{01}/2+(1+e_{\mathrm{m}}^{G})(1-e_{\mathrm{e}}^{01}-\delta_{\mathrm{e}}^{01})/2$	$\delta_{\mathrm{m}}^{G}(1-e_{\mathrm{e}}^{01}-\delta_{\mathrm{e}}^{01})/2+\delta_{\mathrm{e}}^{01}/2$
W/D (11)	w^{11}	0	0	$(1-e_{\mathrm{m}}^{G}-\delta_{\mathrm{m}}^{G})(1-e_{\mathrm{e}}^{11}-\delta_{\mathrm{e}}^{11})/2$	$e_{\mathrm{e}}^{11}+\delta_{\mathrm{e}}^{11}/2+(1+e_{\mathrm{m}}^{G})(1-e_{\mathrm{e}}^{11}-\delta_{\mathrm{e}}^{11})/2$	$\delta_{\mathrm{m}}^{G}(1-e_{\mathrm{e}}^{11}-\delta_{\mathrm{e}}^{11})/2+\delta_{\mathrm{e}}^{11}/2$
W/R	1	1/2	1/2	0	0	0
W/R (10)	w^{10}	$(1-e_{\mathrm{e}}^{10}-\delta_{\mathrm{e}}^{10})/2$	$(1+e_{\mathrm{e}}^{10}+\delta_{\mathrm{e}}^{10})/2$	0	0	0
W/R (01)	w^{01}	$(1-e_{\mathrm{e}}^{01}-\delta_{\mathrm{e}}^{01})/2$	$(1+e_{\mathrm{e}}^{01}+\delta_{\mathrm{e}}^{01})/2$	0	0	0
W/R (11)	w^{11}	$(1-e_{\mathrm{e}}^{11}-\delta_{\mathrm{e}}^{11})/2$	$(1+e_{\mathrm{e}}^{11}+\delta_{\mathrm{e}}^{11})/2$	0	0	0
D/D	0	0	0	0	1	0
D/R	0	0	0	0	1/2	1/2
R/R	0	0	1	0	0	0

ǂ (10), (01), or (11) denotes a mosaic type with effects of deposited parental nuclease from mother, father or both.

Table S3. Model parameters and baseline values.

Symbol	Parameter	Baseline value
e_{m}^{F}, e_{m}^{M}	Meiotic rate of HR in females and males	0.90
δ_{m}^{F}, δ_{m}^{M}	Meiotic rate of EJ repair in females and males	0.05
e_{e}^{10}, e_{e}^{01}, e_{e}^{11}	Embryonic rate of HR with parental effect from mother, father, or both parents	0
δ_{e}^{10}, δ_{e}^{01}, δ_{e}^{11}	Embryonic rate of EJ with parental effect from mother, father, or both parents	0
w^{10}, w^{01}, w^{11}	Female heterozygote fitness with parental effects from mother, father or both*	1
d_0	Initial frequency of transgenic W/D males in male population	0.01

*Fitness reductions due to parental deposition are assumed to affect W/W, W/D and W/R females, unless otherwise specified.
